# A Case of Immunomodulator-Responsive Hypersensitivity Pneumonitis Secondary to Chronic Passive Smoke Inhalation

**DOI:** 10.7759/cureus.58723

**Published:** 2024-04-22

**Authors:** Rabi Bhatta, Jaafar Abou-Ghaida, Sanket Bhattarai, Cyril Blavo

**Affiliations:** 1 Internal Medicine, Universal Health Services, Inc. (UHS) Southern California Medical Education Consortium, Temecula, USA; 2 Osteopathic Medicine, Nova Southeastern University Dr. Kiran C. Patel College of Osteopathic Medicine, Clearwater, USA; 3 Clinical and Translational Medicine, Larkin Health System, South Miami, USA; 4 Pediatrics, Nova Southeastern University Dr. Kiran C. Patel College of Osteopathic Medicine, Clearwater, USA

**Keywords:** ground glass opacity, mycophenolate mofetil, extrinsic allergic alveolitis, chronic interstitial lung disease, hypersensitivity pneumonitis (hp)

## Abstract

Hypersensitivity pneumonitis (HP) is a pulmonary disease characterized by inflammation and fibrosis of the lung parenchyma following chronic exposure to immunogenic antigens. The pathophysiology of HP involves type 3 and type 4 hypersensitivity reactions leading to acute and chronic manifestations, respectively. Clinically, it manifests as exertional dyspnea and wheezing. Pulmonary function tests display a pattern of restrictive lung disease, and high-resolution CT scans display a pattern of ground glass opacities, centrilobular nodules, and mosaic attenuation. Antigen avoidance remains the only method for primary prevention. Alternative therapy may be needed due to either the inability to avoid antigens or the lack of antigen identification. Prednisone 0.5 mg/kg per day is the first-line treatment for acute non-fibrotic forms of HP. In chronic or fibrotic HP, the immunomodulator mycophenolate mofetil (MMF) was shown to be an effective treatment in improving the diffusing capacity of the lungs for carbon monoxide and forced vital capacity, but not overall survival. The following study aims to bring to attention the need for additional prospective multicenter clinical trials to clarify the role of MMF as an immunomodulator in fibrosing HP.

## Introduction

Interstitial lung disease (ILD) is a collection of lung pathologies involving parenchymal tissue that share common clinical, radiographic, and pathological features [1-2]. ILD is sub-classified into idiopathic pulmonary fibrosis, exposure-related pneumonitis, pneumonitis secondary to underlying diseases, and infectious pneumonitis. Exposure-related pneumonitis includes hypersensitivity pneumonitis (HP), pneumoconiosis, substance-related pneumonitis, and radiation pneumonitis. Pneumonitis secondary to underlying diseases includes connective tissue diseases such as systemic sclerosis, rheumatoid arthritis, dermatomyositis, systemic lupus erythematosus, and sarcoidosis. It may also include vasculitis such as granulomatosis with polyangiitis and eosinophilic granulomatosis with polyangiitis. Infection-induced pneumonitis may be caused by *Legionella pneumophila* and *Mycobacterium tuberculosis*.

HP, also described as extrinsic allergic alveolitis, is a subtype of ILD that develops secondary to exposure to inhaled immunogenic antigens [3]. HP categories were previously classified as acute, subacute, and chronic [4]. However, such a classification lacks applicability due to the absence of distinguishable diagnostic criteria. A clinical scenario portraying such limitations is best found in patients who present with fibrosis without experiencing prior symptoms. According to the American Thoracic Society, a new classification categorizing patients into acute inflammatory phase or chronic fibrotic phase is much more reliable in assessing the clinical course and prognosis based on the degree of fibrosis [5]. In the United States, the one-year prevalence rate of HP was estimated at 1.67 to 2.71 per 100,000, with an increase to 11.2 per 100,000 among those aged 65 years and older between 2003 and 2014 [6]. In addition, age-adjusted mortality in the United States showed an increasing trend from 0.12 to 0.68 per million between 1988 and 2016 [7].

The pathophysiology of HP, as the name suggests, involves a hypersensitivity reaction. It is unclear why only a minority of cases with exposure to inciting factors develop this condition. A two-hit hypothesis has been suggested as a possible explanation. It suggests the need for genetic susceptibility followed by environmental antigen exposure for the induction of HP [4]. In terms of pathophysiology, two pathways are thought to play a role in disease progression. In the acute inflammatory phase, a type 3 hypersensitivity reaction involving immune complex deposition and subsequent inflammation is suspected. This is supported by the presence of increased serum IgG titers and lung neutrophils on diagnostic testing [8-9]. Chronic fibrosis, on the other hand, is thought to be due to a type 4 T cell-mediated immune response [9]. Studies have indicated that the involvement of CD4+ cells, seen as an increase in the CD4+/CD8+ ratio, plays a significant role in chronic fibrosis. Specifically, Th17 T cells have been shown to be implicated in this response, as the numbers seem to increase after antigen exposure [9]. Genetic deletion and antibody depletion of IL-17 exert a protective effect on the lungs against fibrosis [4,8-9]. Genetic susceptibility includes primarily polymorphisms in the MHC class II alleles, specifically HLA-DR and DQ [4]. Such polymorphisms are seen in immunoproteasome catalytic subunit b type 8 (PSMB8) and in transporters associated with antigen processing (TAP), which are typically responsible for breaking down and loading antigens on MHC class I molecules, respectively [4].

Suspicion for HP and subsequent workup starts with a thorough history-taking and physical examination. The history of exposure is particularly important. Medications, such as amiodarone and bleomycin, bird feathers, droppings, humidifiers, and ceiling mold, are some of the many exposures linked to HP. Clinical signs of HP are usually nonspecific, and more common diseases, such as chronic obstructive pulmonary disease, must be ruled out first. Patients with HP, especially the chronic forms, are likely to exhibit a gradual onset of exertional dyspnea, fatigue, cough, sputum production, finger clubbing, and weight loss [10]. On auscultation, bibasilar crackles may be heard with accompanying inspiratory wheezing in cases of coexisting bronchiolitis [10].

Pulmonary function tests (PFT) may be used to assess disease severity, progression, or resolution with therapy. Imaging with chest X-rays is of limited significance when diagnosing HP. Twenty percent of cases are negative, especially in acute forms of HP [11]. Common findings on chest X-rays include ground glass or reticulonodular opacities that spare the lung bases. High-resolution CT (HRCT), on the other hand, is useful in differentiating the clinical forms of HP. In the acute form, ground glass opacities are the predominant feature, with or without centrilobular nodules. In the chronic fibrotic form, patchy ground glass attenuation, centrilobular nodules, and mosaic attenuation, also known as the “three density pattern” or “head cheese sign,” are seen in a lobular distribution and are highly specific findings [12]. Lung biopsies in HP may reveal histological features such as chronic interstitial pneumonitis with bronchial cellular infiltrate, chronic bronchiolitis, or non-necrotizing granulomatous inflammation of the peribronchial interstitium [13].

Therapies to treat ILD include corticosteroids, mycophenolate mofetil (MMF), azathioprine, methotrexate, cyclophosphamide, and rituximab [14]. Corticosteroids are the first-line therapy for the treatment of ILD [14]. In the context of HP, prednisone is effective in treating inflammatory non-fibrotic HP by increasing forced vital capacity (FVC), but it has proved less effective against fibrosing HP [14]. Typically, the initial prescribed dosage for prednisone is 0.5 mg/kg per day (with a maximum of 30 mg per day) for a period of four to eight weeks, after which the dose is gradually reduced to 10 mg per day within three months [15]. The clinical effects of the aforementioned therapies have also been studied in fibrosing HP. The purpose of our study is to discuss mycophenolate's beneficial clinical effect, its limitations, and the need for more prospective clinical trials to better understand its role as an immunomodulator in fibrosing HP.

## Case presentation

The patient is a 73-year-old Nepalese female with chief complaints of progressive intermittent shortness of breath and nonproductive cough for the past three years. She reported exertional dyspnea and diaphoresis exacerbated by walking up a flight of stairs or uphill and relieved with rest. The physical evaluation is positive for audible wheezing with exertion and rest. She tried over-the-counter antitussive medication. However, it did not improve her cough. She is a non-smoker and lives with relatives. She has a history of exposure to biomass smoke for over 20 years and lives in moldy and damp rooms. She spends four to five hours every day in a poorly ventilated kitchen cooking food on a wooden stove and admits to prolonged smoke exposure. Other symptoms include bilateral lower extremity swelling, nocturnal calf muscle claudication, and muscle twitching for the past three years. She has a past medical history significant for a self-limited flu-like illness consistent with COVID-19 during the peak of the 2019 pandemic, for which she did not receive any screening or diagnostic tests. The patient denies symptoms of recurrent fevers, night sweats, hemoptysis, swollen glands, weight loss, paroxysmal nocturnal dyspnea, orthopnea, bendopnea, shortness of breath, palpitation, and chest pain, making infectious and cardiovascular etiologies less likely.

On physical examination, there were no signs of clubbing or jugular venous distention. Cardiac auscultation revealed normal heart sounds without murmurs. Lung auscultations revealed fine crepitation on prolonged expiration. Lower extremities showed mild pitting edema bilaterally, without signs of swelling or redness, indicating a possible cor pulmonale given the possible respiratory symptoms. Routine CBC and comprehensive metabolic panel were within normal limits. The patient’s oxygen saturation was 92% on room air, and respiratory rate was elevated at 20 breaths per minute. A chest X-ray demonstrated calcified lymph nodes in the right lower lobe with prominent pulmonary markings. The echocardiogram was suggestive of normal systolic function with a left ventricular ejection fraction of 60% and concentric left ventricular hypertrophy with grade I left ventricular diastolic dysfunction. This rules out heart failure as a possible cause of shortness of breath.

**Figure 1:**
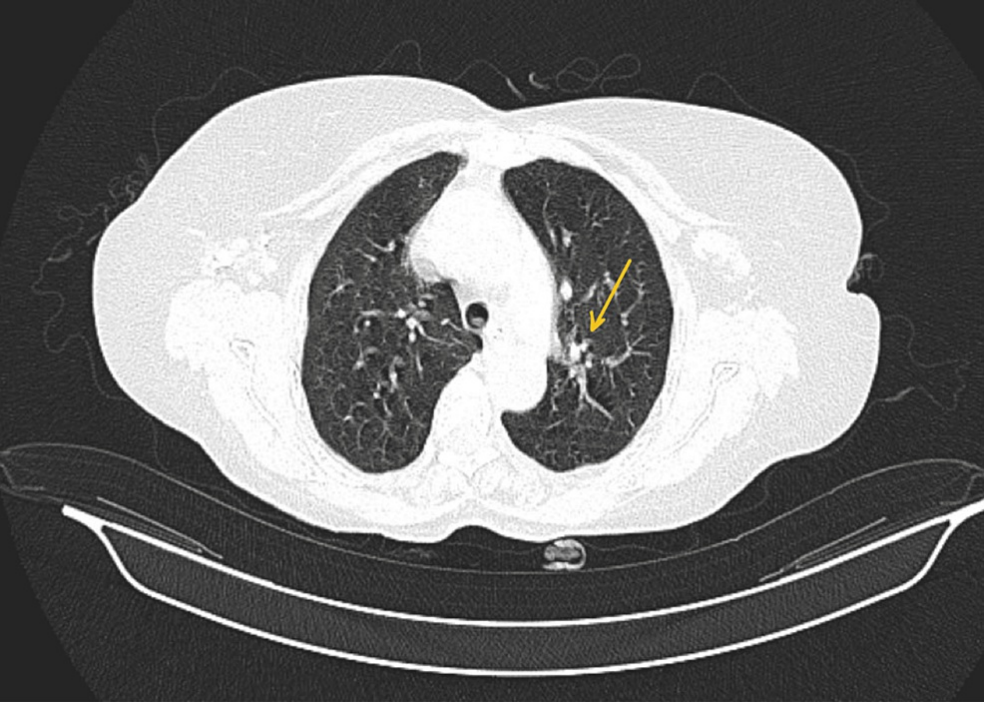
Pre-treatment HRCT showing ground glass opacities in the posterior basal segment of the left lower lobe, subsegmental atelectasis in the inferior lingular segment of the left upper lobe (yellow arrow), and fibrotic changes in the left lower lobe HRCT: High-resolution computed tomography

**Figure 2:**
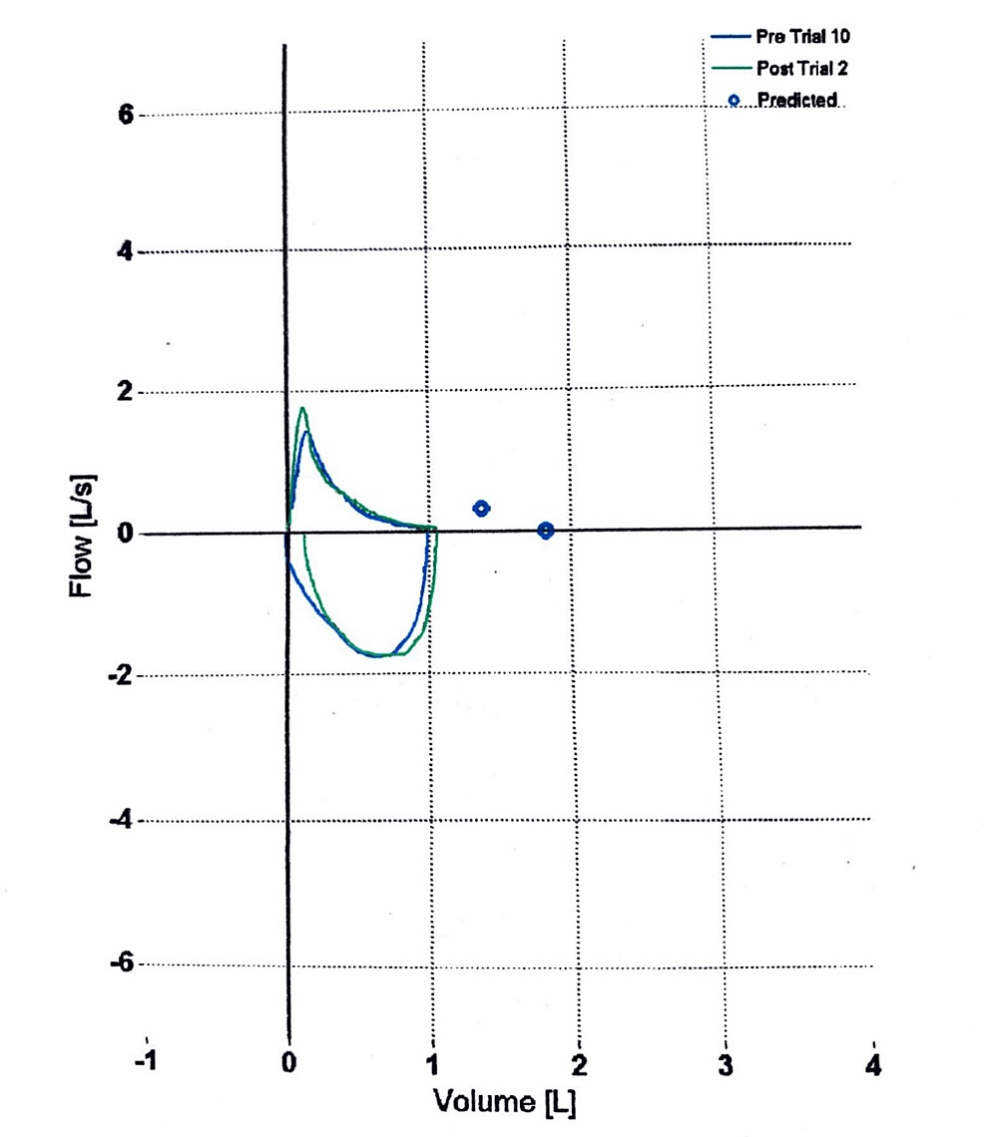
Pre-treatment volume-flow curve displaying a mixed pattern of emphysematous lung disease seen in the characteristic scooped-out curve and restrictive fibrosis seen as a reduced total lung capacity totaling 1 liter before bronchodilator treatment (blue graph). A modest increase in expiratory flow rate is observed after bronchodilator treatment (green graph) Flow (L/s): flow (liters/seconds), volume (L): volume (liters)

**Figure 3:**
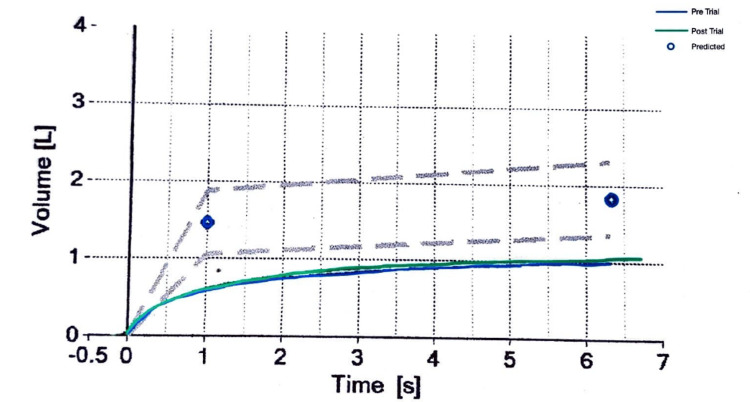
Pre-treatment PFT function test depicting the volume of inspired over time (blue line) with limited increase following bronchodilator therapy (green line) PFT: pulmonary function test, volume (L): volume (liters), time (s): time (seconds)

Chest HRCT revealed consolidation, ground glass opacities, subsegmental atelectasis, and fibrotic changes (Figure 1). Enlarged and calcified lymph nodes in both axillae were present. PFT included spirometry and peak flow measurement, which showed severe restrictive airway disease with no significant post-bronchodilator reversibility (Figure 2) and reduced total lung capacity (Figure 3). As a result, the patient was initially treated symptomatically and prescribed prokinetics, pantoprazole, doxycycline, a bronchodilator, and expectorants.

**Figure 4:**
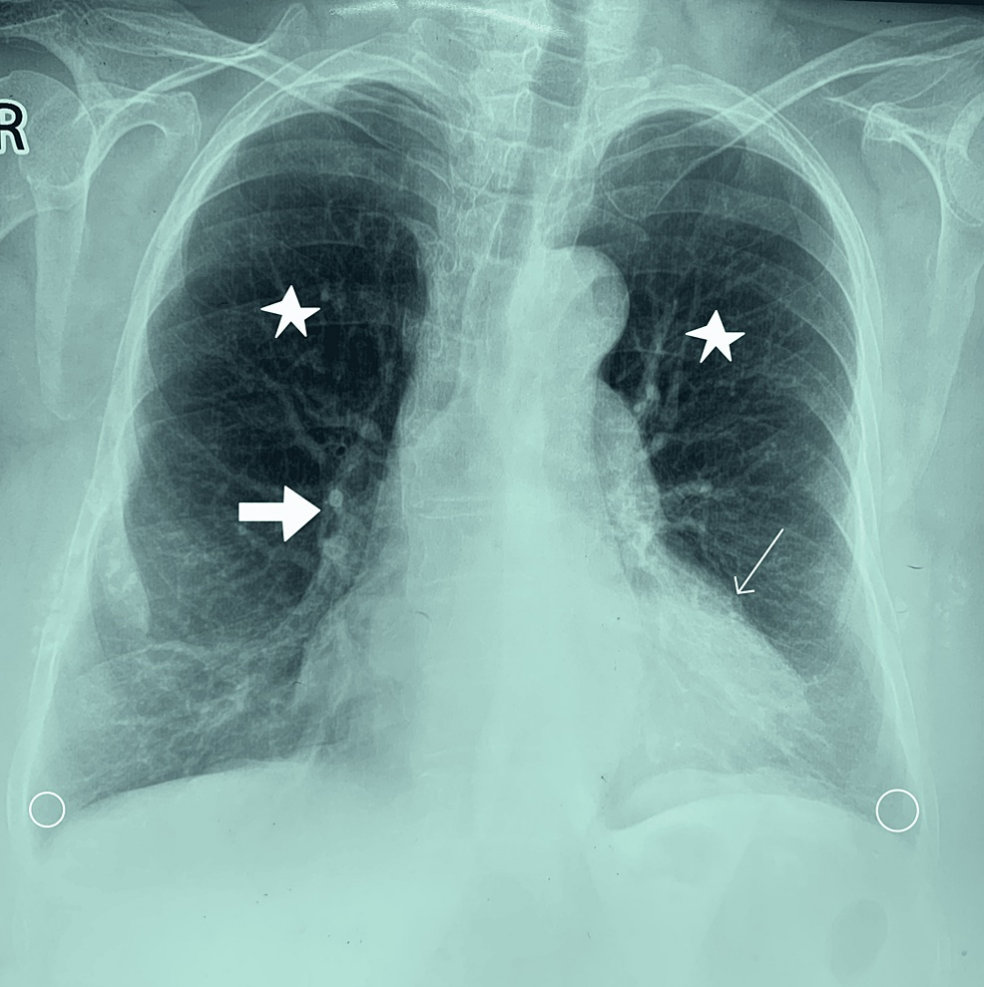
Post-treatment chest X-ray revealing clear lung fields (star) with normal bronchovascular markings (thick arrow). Costophrenic angles (circles) and heart size (thin arrow) are normal

During the first follow-up, one month after the initial visit, the patient still complained of shortness of breath and nocturnal dry cough. On lung auscultation, crepitation was noted bilaterally. After a six-minute walk test, SpO2 at room air was 75% and heart rate was 100 bpm. A tuberculin skin test was ordered, which was non-reactive. Based on her clinical progression, laboratory workup, and radiological findings, a diagnosis of ILD was made. The patient was prescribed MMF 500 mg twice a day, along with a combination of an inhaled corticosteroid and a bronchodilator. On the second follow-up visit, two months after the first follow-up, the patient reported remarkable improvement in her symptoms, and lung auscultation revealed scanty crepitations with prolonged expiration. A post-treatment chest X-ray revealed normal bronchovascular markings (Figure 4). In addition, she reported moving to a less humid and properly ventilated apartment prior to the second follow-up visit. MMF was tapered to 500 mg once daily for another two months. On the third follow-up, two months after the second follow-up visit, the patient reported marked improvement in coughing and shortness of breath. A diagnosis of HP was subsequently made. The patient tolerated the treatment without any adverse outcomes and has demonstrated significant clinical improvement with continued medical follow-up. Unfortunately, due to cost limitations, post-treatment HRCTs and PFTs were not conducted.

## Discussion

HP is a subtype of ILD that develops secondary to inhaled immunogenic antigens. It is a mixture of type 3 hypersensitivity, leading to acute inflammatory changes, and type 4 hypersensitivity, leading to chronic fibrosis. In this patient, a clear set of environmental allergens were identified on HPI. The patient admitted to being exposed to a burning wood stove, ceiling mold, and a poorly ventilated apartment for approximately 20 years. This translates to three possible exposures that can explain how this patient developed HP. It also justifies the adoption of a multifactorial approach to her treatment. One that includes both antigen avoidance and immunosuppressive therapy. MMF’s promising effects in retrospective clinical trials and our patient’s case justify its adoption as a first-line therapy for fibrosing HP.

Studies have demonstrated that MMF improves the diffusing capacity of the lungs for carbon monoxide (DLCO) and stabilizes FVC while being associated with less toxicity and treatment-related adverse events [14]. A retrospective study by Morisset et al. revealed that, in a cohort of 70 patients, 51 treated with MMF, treatment resulted in a nonsignificant increase in FVC by 1.3% and a statistically significant increase in DLCO by 3.9% after one year of treatment [16]. Another retrospective longitudinal analysis by Adegunsoye et al. revealed that, in a cohort of 131 patients treated with combination therapy of MMF and prednisone, the MMF/prednisone combination resulted in a reduced incidence of treatment-emergent adverse events (incidence rate 0.075) compared to the prednisone-only group (incidence rate 0.198) [17]. Mycophenolate was demonstrated as an effective treatment option for chronic HP, which also decreased the daily prednisone dosage without jeopardizing FVC [16-17].

Due to resource limitations in Nepal, imaging and PFTs had to be ordered conservatively. In this case, HRCT and PFTs were ordered once to obtain both high-quality imaging of the lungs and study lung physiology, which helped clinch the diagnosis of HP. On follow-up, a post-treatment chest X-ray was ordered instead of PFTs to track the overall progression of the disease after mycophenolate therapy. The administration of MMF has resulted in significant clinical and radiological improvement and no adverse effects. Clinically, the patient reports reduced wheezing and exertional dyspnea. Chest radiographs have revealed significant improvement in pulmonary markings. Antigen avoidance may have further amplified the clinical effects of the therapy. This combined treatment approach not only improved the patient’s respiratory symptoms and reduced the overall burden of the disease, but also confirmed the diagnosis of HP.

Even though studies have concluded that MMF improves PFT readings, they have not introduced any benefit to overall survival [17]. This may be attributed to either the disease severity at the time of the study, the progressive nature of the disease, specific patient phenotypes, or the role of immunosuppression with corticosteroids [17]. Additional prospective multicenter clinical trials are needed to clarify the role of MMF as an immunomodulator in fibrosing HP, but its beneficial effect in clinical trials and individual patient cases maintains its first-line status for fibrosing HP.

## Conclusions

HP is a subtype of ILD and an allergic reaction triggered by a number of chronic environmental exposures. Cellular processes in HP lead to inflammation and subsequent fibrosis involving T and B cells engaging in a mixture of type 3 and type 4 hypersensitivity reactions. Highlights of the clinical symptoms of this patient were exertional dyspnea and reduced exercise tolerance. The diagnostic test revealed a mixed pattern of obstructive and restrictive pulmonary disease. PFT studies demonstrated reduced total lung capacity. HRCT revealed consolidation, ground glass opacities, subsegmental atelectasis, honeycomb appearance, and fibrotic changes. The use of MMF resulted in significant improvement in the patient’s clinical symptoms and radiographic findings. This outcome suggests that MMF can be used as a treatment option for patients with fibrosing HP. Retrospective studies have shown that mycophenolate improves FVC and DLCO, but not overall survival. Our study proves the need for additional prospective clinical trials to better clarify the role of MMF.
